# Single‐cell sequencing reveals alterations in the differentiation of bone marrow haematopoietic cells in patients with paroxysmal nocturnal haemoglobinuria

**DOI:** 10.1002/ctm2.1671

**Published:** 2024-06-25

**Authors:** Hui Liu, Wei Wang, Chaomeng Wang, Liyan Li, Junshu Wu, Yingying Chen, Zhaoyun Liu, Honglei Wang, Lijuan Li, Rong Fu

**Affiliations:** ^1^ Department of Hematology Tianjin Medical University General Hospital Tianjin People's Republic of China; ^2^ Shanghai Institute for Advanced Immunochemical Studies and School of Life Science and Technology ShanghaiTech University Shanghai People's Republic of China

Dear Editor,

Paroxysmal nocturnal haemoglobinuria (PNH) is a rare acquired haematopoietic stem cell (HSC) clonal disorder primarily linked to a somatic mutation in the phosphatidylinositol glycan class A (PIGA) gene located on the X chromosome. These somatic mutations result in incomplete glycosyl‐phosphatidylinositol (GPI)‐anchored protein synthesis, which activates the complement system and leads to clinical manifestations like intravascular haemolysis, bone marrow (BM) failure and thrombosis.^1^ CD59, also known as membrane attack complex inhibition factor, protects host cells from complement system attacks and lysis. Due to PIG‐A gene mutation, various GPI‐anchored proteins on the cell membrane, such as CD59, are missing. Therefore, the detection of CD59 is the gold standard for diagnosing PNH. Immune escape, anti‐apoptosis and secondary gene mutation mechanisms contribute to the development of PNH.^2^, ^3^ However, the proliferation and differentiation of BM haematopoietic cells in PNH remain incompletely understood.

In our study, we obtained BM mononuclear cells from classical PNH patients and healthy controls (*n* = 4 each; clinical data are shown in Table 1). We sorted CD59^+^ and CD59^−^ cells using flow cytometry and performed deep single‐cell RNA sequencing on all haematopoietic cell lines (Figure 1A). We conducted an initial analysis to compare normal BM haematopoietic cells (CD59^+^ cells, P group) with PNH clones (CD59^−^ cells, N group) in PNH patients, as compared to healthy controls (C group).

**TABLE 1:** The clinical characteristics of four patients with paroxysmal nocturnal haemoglobinuria.

Characteristics	Patient 1	Patient 2	Patient 3	Patient 4
Sex	Female	Female	Female	Female
Age (years)	30	18	35	50
Hb (g/L)	65	59	63	62
Ret (%)	22.28	7.89	11.72	15.68
TBIL/IBIL (umol/L)	77.5/65	21.5/17.6	23.3/17.3	49/31.8
LDH (U/L)	3407	1548	1957	2790
Platelet (× 10^9^/L)	101	118	153	62
Neutrophil (× 10^9^/L)	2.06	1.55	1.11	1.37
CD59^−^ granulocyte (%)	83.53	98.33	54.42	95.01
CD59^−^ RBC (%)	II 14.03, III 21.09	II 3.27, III 9.08	II .25, III 9.26	II 3.37, III 50.83
Flaer‐ granulocyte (%)	90.27	96.83	61.36	97.69
Bone marrow biopsy	MEH	MEH	MEH	MEH
D‐Dimer (ng/mL)	1140	1140	834	879
Creatinine (umol/L)	30	31	45	35
Diagnosis	Classical PNH	Classical PNH	Classical PNH	Classical PNH
Therapy	Sodium bicarbonate, vitamin E, RBC transfusion	Glucocorticoids, immunosuppressants, RBC transfusion	Glucocorticoids, RBC transfusion	Glucocorticoids, cyclosporin A, anticoagulants, RBC transfusion
Time from diagnosis (year)	3	2	15	5
Thrombosis	–	–	–	Portal vein thrombosis
Comorbidity	Depression	–	–	–

Abbreviations: Hb, haemoglobin; IBIL, indirect bilirubin; LDH, lactic dehydrogenase; RBC, red blood cells; Ret, reticulocyte; TBIL, total bilirubin; PNH, paroxysmal nocturnal haemoglobinuria.

**FIGURE 1:**
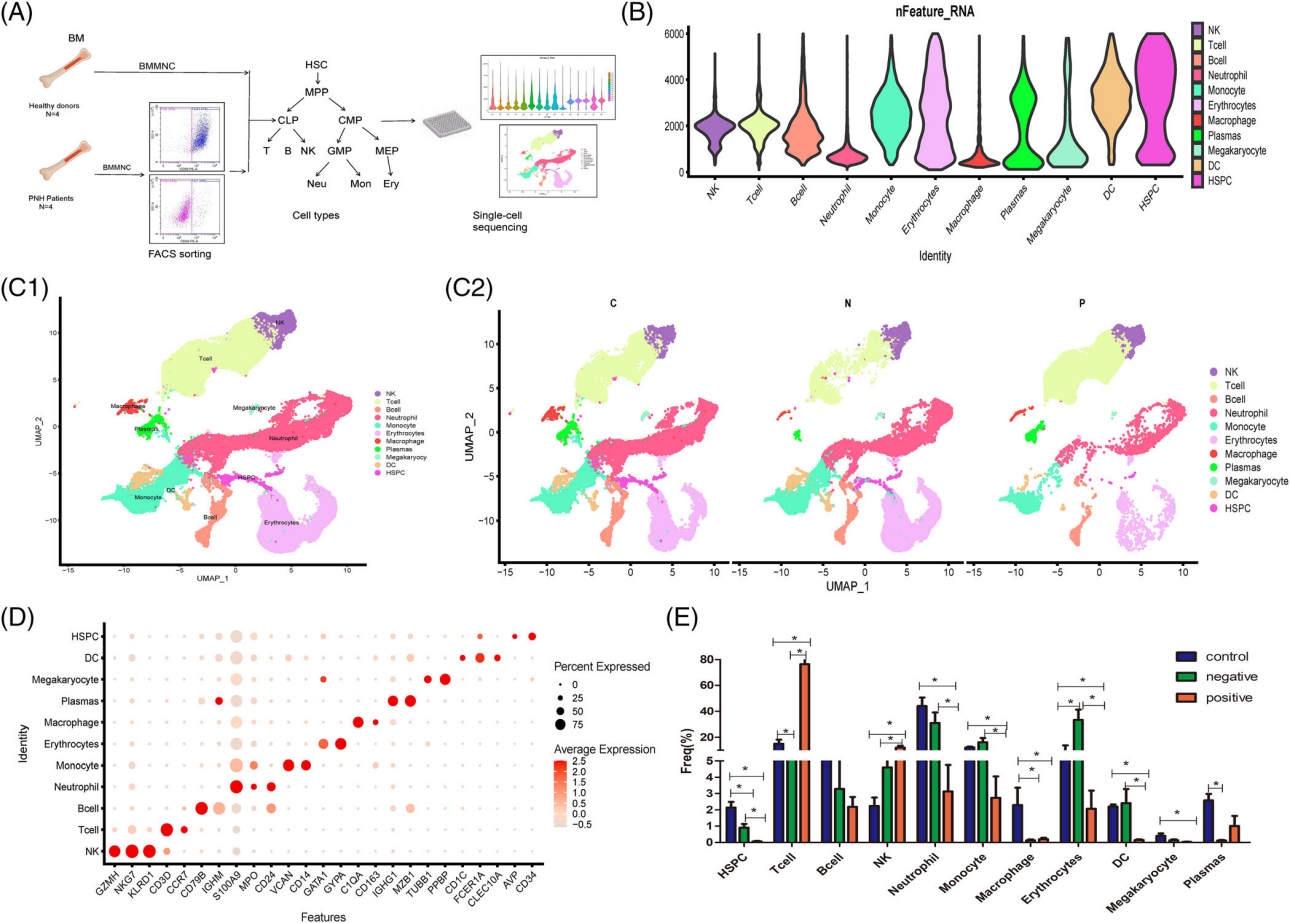
Haematopoietic cell differences in bone marrow between patients with paroxysmal nocturnal haemoglobinuria (PNH) and healthy controls. (A) Experimental design of bone marrow cell single cell sequencing. (B) Violin plots showing the number of detected genes in each cell type. (C) UMAP distribution of haematopoietic cells in bone marrow (C1), and the differences between patients with PNH (N and P groups) and healthy controls (C2). (D) Dot plot of cell‐specific markers. The size of the dot represents the percentage of cells expressing the markers, and the colour encodes the average scaled expression values. (E) The ratio of each observed cell type in the PNH patients and normal controls. CLP, common lymphoid progenitors; CMP, common myeloid progenitors; GMP, granulocyte‐monocyte progenitors; HSC, haematopoietic stem cell; HSPC, haematopoietic stem and progenitor cell; MEP, megakaryocyte‐erythrocyte progenitors; UMAP, uniform manifold approximation and projection.

We sequenced a total of 10 705 cells (ranging from 5515 to 14 162) from each sample, with an average of 1419 genes analyzed per cell. Our analysis revealed 11 clusters, including haematopoietic stem and progenitor cells (HSPCs), neutrophils, lymphocytes (B cells, T cells, NK cells and plasma cells), monocytes, macrophages, dendritic cells (DCs), megakaryocytes and erythrocytes (Figure 1C). We identified specific markers associated with each cell type, such as CD34, CD79B, GZMH, CD3D, S100A9, VCAN, GATA1 and PPBP (Figure 1D).

We found that the proportions of haematopoietic cells in various lineages in PNH patients have changed (Figure 1E), which has been confirmed by flow cytometry (Figure S1). The proportion of HSPCs in PNH patients was significantly lower than in healthy controls, indicating impaired HSCs, particularly a reduced number of normally cloned HSPCs leading to varying degrees of pancytopenia. In lymphoid lineage differentiation, we observed a significant increase in T and NK cells in PNH patients, primarily driven by normal clones, whereas B and plasma cells were significantly decreased. We further performed differential expressed genes (DEGs) analysis in T and NK cells. In T cells, compared with controls, the upregulated genes (TNFAIP3, IRF1, DDX3X, PIK3R1, JUN and PTPRC) in P group indicate the enrichment in type I interferon production and the activation of T cell receptor signalling pathway; compared with N group, the expressions of IL7R, IGLC2, ANXA1, IGKC and IGHA1 genes upregulated the activation of lymphocyte (Figure S2 and Table S1). In NK cells, the downregulated genes such as RPS4Y1, RPS29, RPS27, RPL38 and RPL23A in N group indicated that CD59^−^ NK cells may have intrinsic defects; while the upregulated genes (ITGB1, ZNF683, LGALS1 and RIPOR2) in P group indicated the activation of CD59^+^ NK cells. Compared with N group, the expression of genes (KLRC2, IGLC2, CXCR4, VIM, CCL5 and ITGB1) upregulated in P group, indicates enhanced immune regulation function of CD59^+^ NK cells (Figure S3 and Table S2).

In myeloid lineage differentiation, we observed a significant reduction in granulocytes and monocytes in normal clones from PNH patients, with no significant difference between PNH clone and normal control. A similar trend was observed in DCs. Macrophages in PNH patients decreased significantly in both normal and PNH clones. Erythroid lineage differentiation revealed that BM erythroid cell proliferation in PNH patients is primarily driven by PNH clone. The analysis of DEGs showed that the genes involved in cell cycle such as IFI27, MKI67 and TERF2IP, and the genes involved in DNA metabolism such as ALAS2, HMBS and UROD were significantly upregulated in N group. Interestingly, we also found some DEGs involved in immune regulation, such as upregulated RSAD2 gene in N group, upregulated CCL5 and IL‐32, downregulated CD74 in P group (Table S3). These results revealed that erythroids may be involved in immune activation, especially T cell activation (Figure S4).

To further investigate changes in HSPCs, we analyzed HSPC differentiation across eight clusters, including HSC, common myeloid progenitors, granulocyte‐monocyte progenitors (GMP), megakaryocyte‐erythrocyte progenitors (MEP), common lymphoid progenitors (CLP), B‐NK cell progenitors (BNK), eosinophil/basophil/mast cell progenitors (EBM) and multi‐lymphoid progenitors (Figure 2A,B). The number of HSCs was significantly lower in both normal and PNH clones from PNH patients. Compared to healthy controls, there were no significant differences in the number of CLP, BNK, GMP, MEP and EBM in PNH clone. However, in the normal clone of PNH patients, all HSPCs were significantly reduced (Figure 2C). Meanwhile, we also conducted enrichment analysis of DEGs in HSPCs (Figure 2D,E and Table S4), and found that the apoptosis of CD59^+^ stem cells was significantly increased in PNH patients, compared with PNH clone and normal controls. These results align with the findings of Chen et al. who reported that PNH clone evades immune attack and destruction, while normal cells experience a stress response followed by programmed cell death.^4^ Interestingly, immune cells such as T cells, DC cells and NK cells were activated in CD59^+^ HSCs of PNH patients (Figure 2F–I).

**FIGURE 2:**
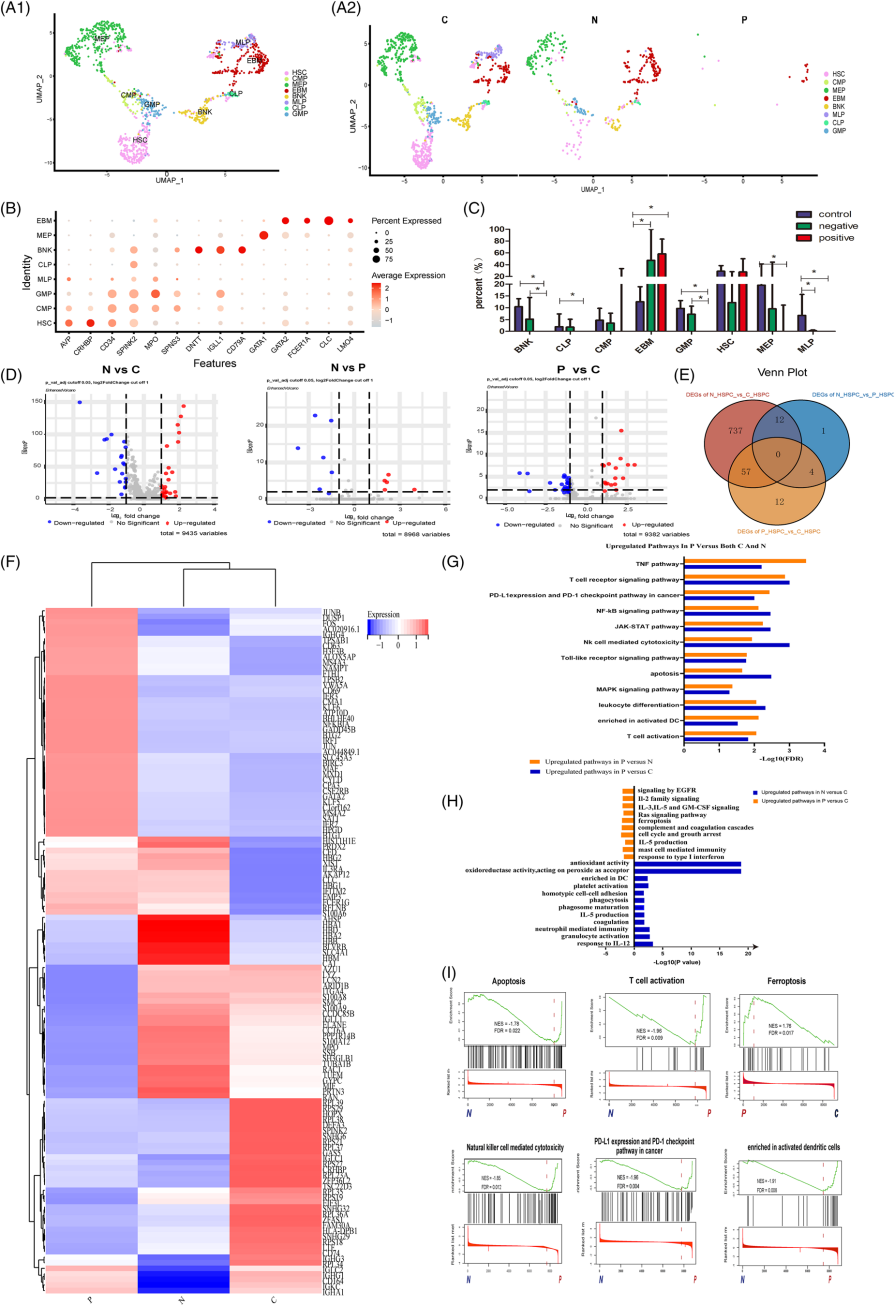
Abnormalities of haematopoietic stem and progenitor cells in paroxysmal nocturnal haemoglobinuria (PNH) patients. (A) UMAP distribution of haematopoietic stem and progenitor cells (HSPCs) (haematopoietic stem cell, common lymphoid progenitor, common myeloid progenitor, granulocyte‐monocyte progenitor, multilymphoid progenitor, megakaryocyte‐erythrocyte progenitor, B‐NK cell progenitor and eosinophil/basophil/mast cell progenitor) (A1), and the differences between patients with PNH (N and P groups) and healthy controls (A2). (B) Dot plot of cell‐specific markers. The size of the dot represents the percentage of cells expressing the markers, and the colour encodes the average scaled expression values. (C) The numbers of each HSPC subtype within each group, ^*^ *p* < 0.05. (D) Volcano plot showing differentially expressed genes (DEGs) of HSPCs in the three groups. (E) Venn diagram showing the summary of DEGs detected by pair‐wise comparison at three groups. (F) Heatmap displaying the expression of some DEGs of HSPCs in the three groups based on significance and log FC values. (G) Functional enrichment analysis of DEGs in HSPCs. Upregulated pathways in P group compared with N and C groups. (H) Upregulated pathways in P and C groups compared with C group. (I) GSEA of genes in P group compared with N group and C group. GSEA, gene set enrichment analysis.

To further investigate the types of cells involved in immune escape and their modes of action in PNH, we performed an unbiased cell–cell interaction analysis using CellChat across all cell lineages. Interaction strength was enhanced in P and N groups (Figure 3A). Additionally, we analyzed signalling pathways with higher combined interaction strength ratios in the different groups (Figure 3B,C). We found that in the SN signalling pathway, SIGLEC1_SPN, which serves as a surrogate marker for activation of the IFN‐I pathway,^5^ was significantly enhanced in both P and N groups (Figure 3D). The ICOS signalling pathways,^6^ ICOSL_ICOS, ICOSL_CD28 and ICOSL_CTLA4, were significantly enhanced in the P group (Figure 3E). The CD46 signalling pathways,^7^ CD46_JAG1, CD70: CD70_CD27,^8^ PVR: PVR_CD226, PVR_TIGIT^9^ and ANGPT1: ANGPT1_TEK, were significantly enhanced in the P group (Figure 3F). The FASLG signalling pathways, FASL_FAS and CD34: CD34_SELP, were significantly enhanced in the N group.

**FIGURE 3:**
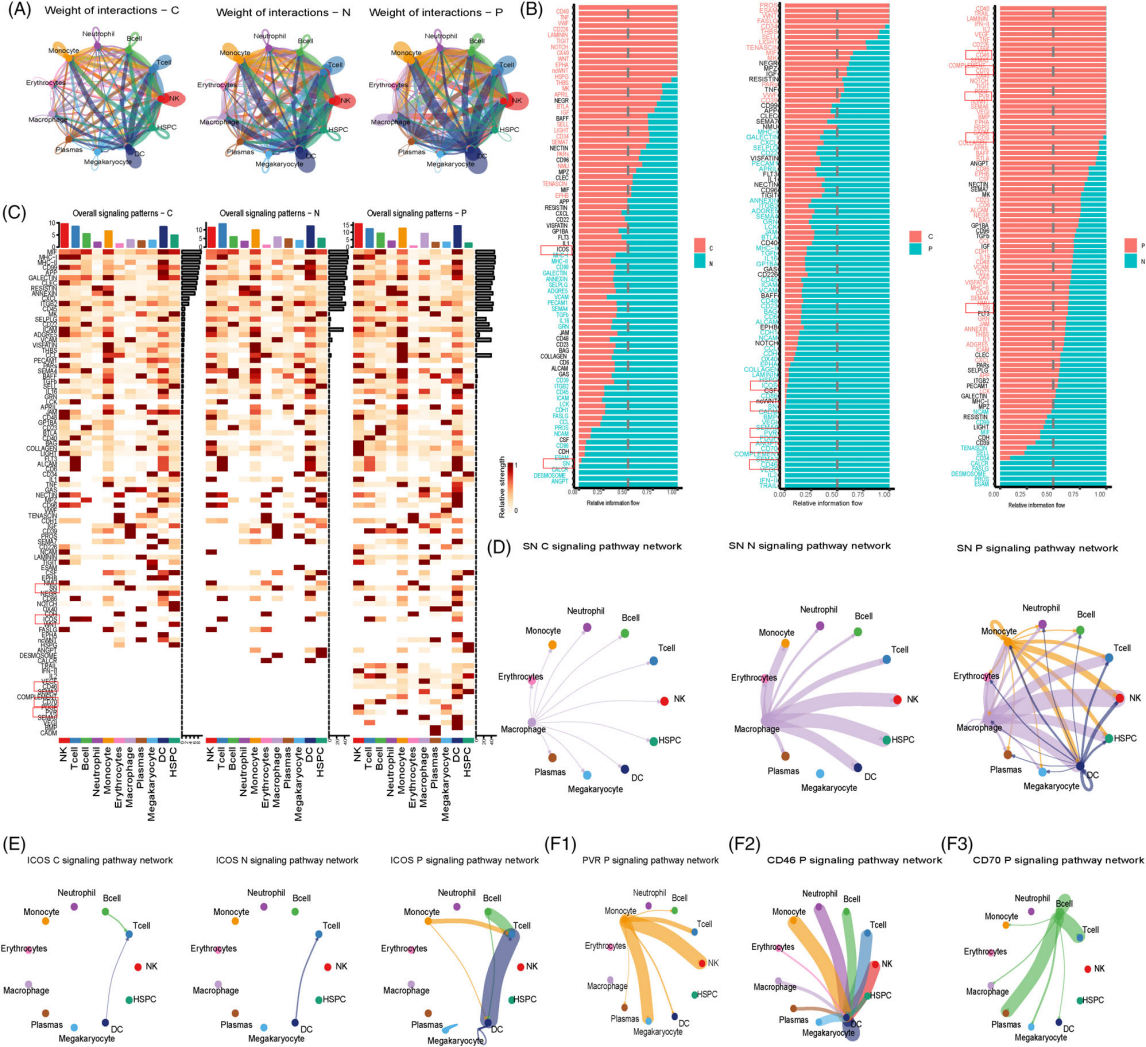
Identification of major signalling changes in paroxysmal nocturnal haemoglobinuria (PNH) by the interaction inference analysis. (A) The interaction strength of the inferred cell–cell communication networks in each of the three groups. (B) Normalized interactions were summed as the overall information flow and significantly different signalling pathways were identified. (C) The overall signalling patterns among our defined cell clusters among the three groups. (D–F) The inferred SN:SIGLEC1_SPN (D), ICOS (ICOSL_ICOS, ICOSL_CD28, ICOSL_CTLA4) (E), PVR: PVR_CD226 and PVR_TIGIT (F1), CD46: CD46_JAG1 (F2), CD70: CD70_CD27 (F3) signalling network among the cell populations represented by the nodes. The edge width represents the interaction strength of the specific pathway.

In conclusion, this is the first study to confirm that the HSPCs are transcriptomically primed for apoptosis, especially CD59^+^HSPCs, and predicted to have altered distributions of haematopoietic cells in PNH. Erythroid cell proliferation is significantly upregulated in CD59^−^ clone. Conversely, in CD59^+^ cells, T and NK cells are significantly increased. The collective involvement of the mononuclear phagocytic system, along with activated normal T and NK cells, in the immune escape of PNH leads to target cell damage.

## AUTHOR CONTRIBUTIONS

Rong Fu designed the study and revised the manuscript. Hui Liu and Wei Wang analyzed the data and wrote the manuscript. Chaomeng Wang, Liyan Li, Junshu Wu, Yingying Chen, Zhaoyun Liu, Honglei Wang and Lijuan Li contributed to patient data collection. All the authors have read and approved the final version of this manuscript.

## CONFLICT OF INTEREST STATEMENT

The authors declare no conflicts of interest.

## FUNDING INFORMATION

This work was supported by the National Natural Science Foundation of China (grant nos. 82270142, 82000128, 81970115 and 81770110), Tianjin Municipal Natural Science Foundation (grant no. 18JCYBJC27200), Tianjin Municipal Commission of Education Research Project (grant no. 2022KJ236) and Tianjin Key Medical Discipline(Specialty) Construction project (grant no. TJYXZDXK‐028A).

## ETHICS STATEMENT

This study was approved by the Ethics Committee of the Tianjin Medical University General Hospital (Ethical no. IRB2024‐YX‐041‐01).

## Supporting information

Supporting Information

Supporting Information

Supporting Information

Supporting Information

Supporting Information

Supporting Information

Supporting Information

Supporting Information

Supporting Information

Supporting Information

## Data Availability

All relevant data are included within the paper.
