# Supplementary figures and images for: Single‐cell sequencing reveals alterations in the differentiation of bone marrow haematopoietic cells in patients with paroxysmal nocturnal haemoglobinuria
Source: Clin Transl Med. 2024 Jun 25;14(7):e1671. doi: 10.1002/ctm2.1671 (PMC11199056; doi:10.1002/ctm2.1671)

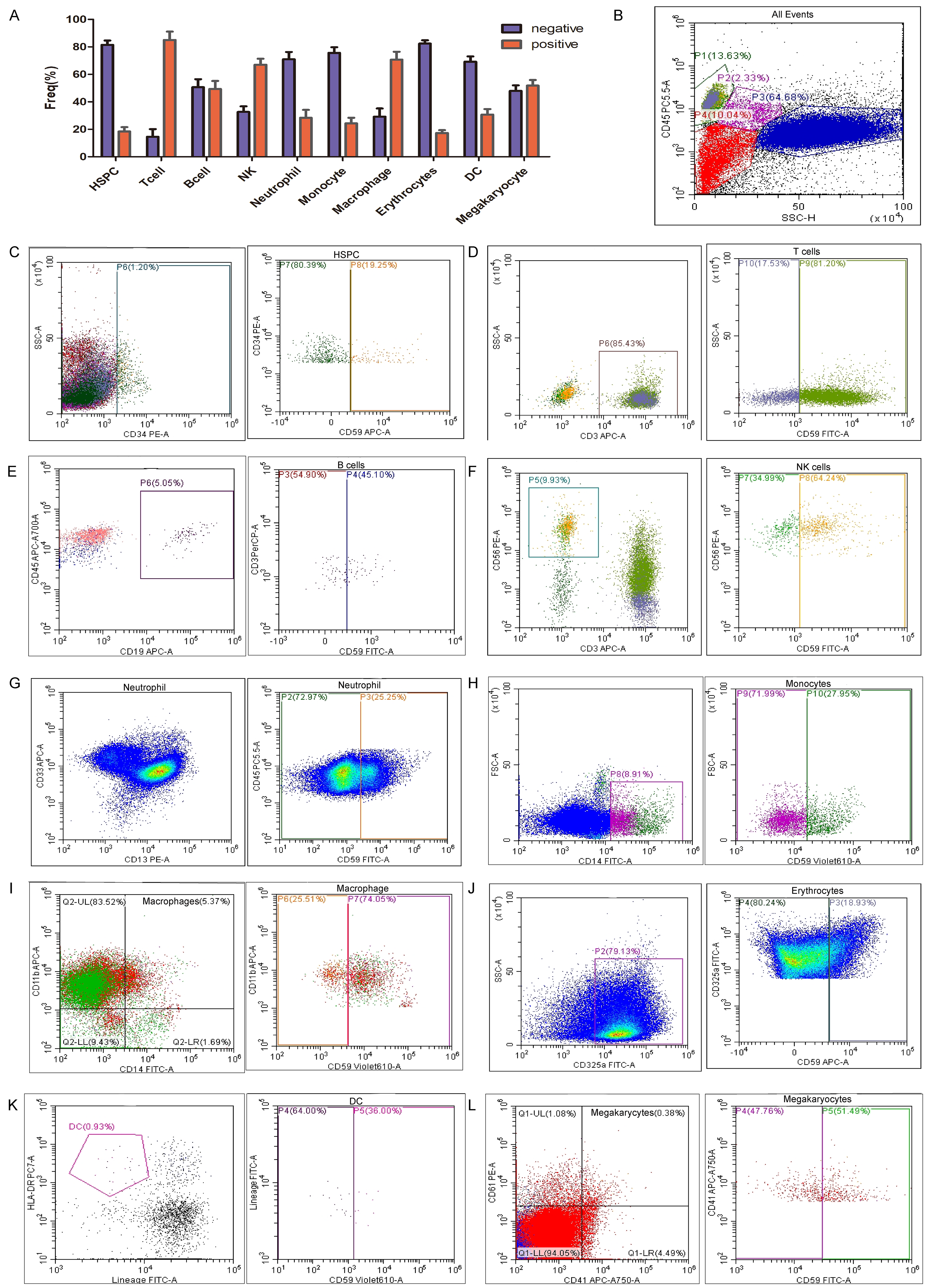

Supplement: Supplementary file 1 — Supporting Information [file CTM2-14-e1671-s002.tif]

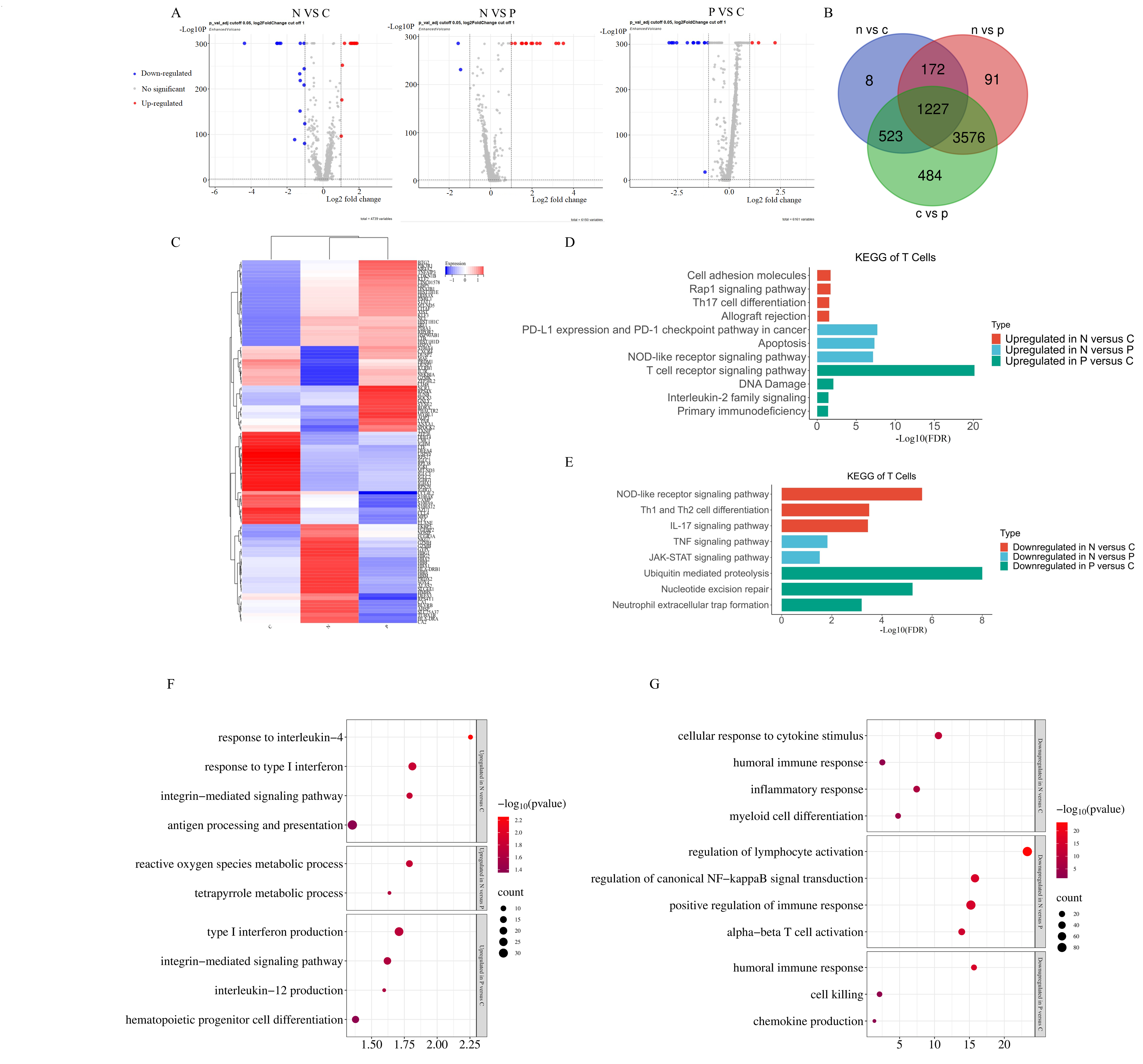

Supplement: Supplementary file 2 — Supporting Information [file CTM2-14-e1671-s010.tif]

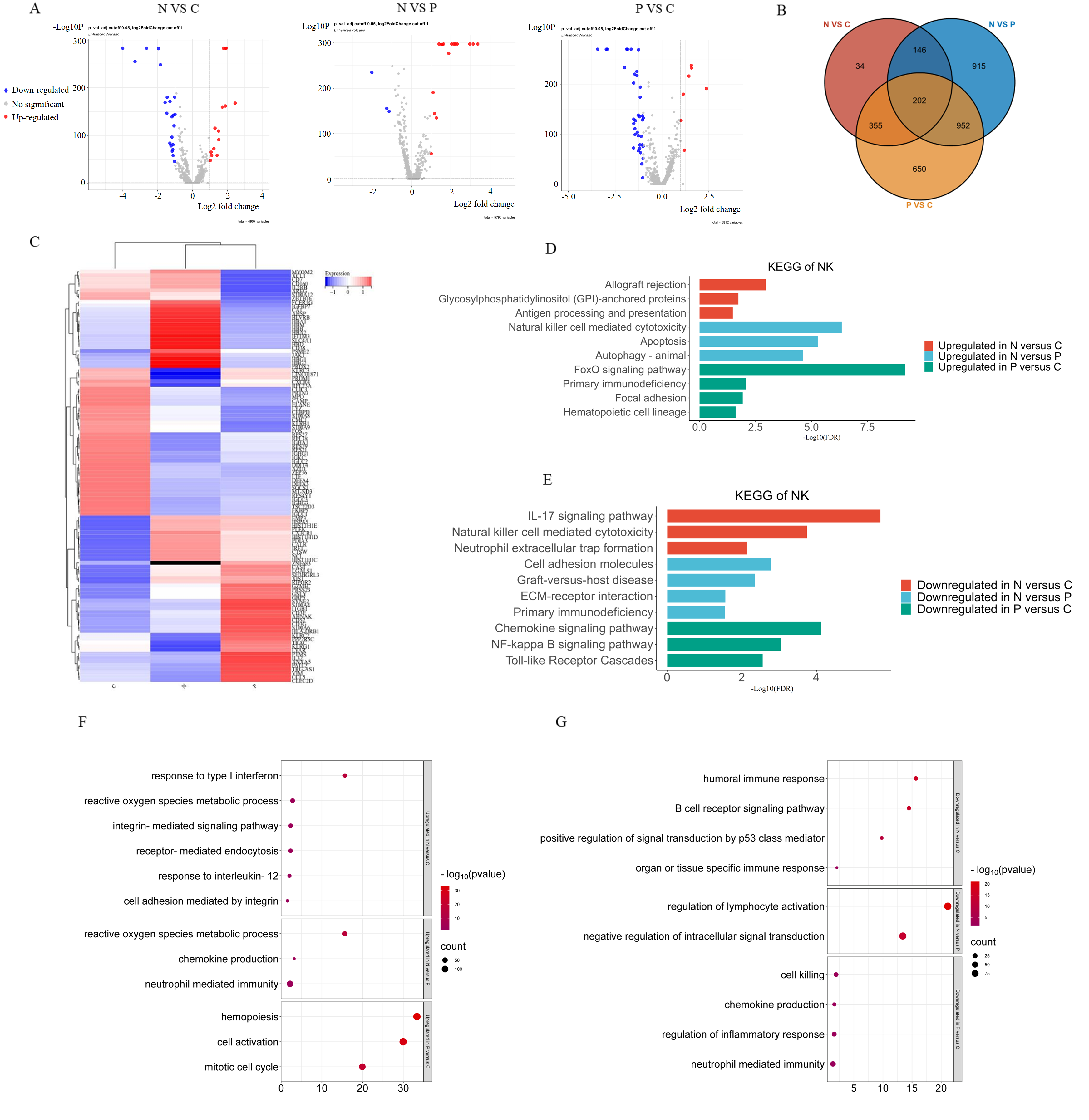

Supplement: Supplementary file 3 — Supporting Information [file CTM2-14-e1671-s009.tif]

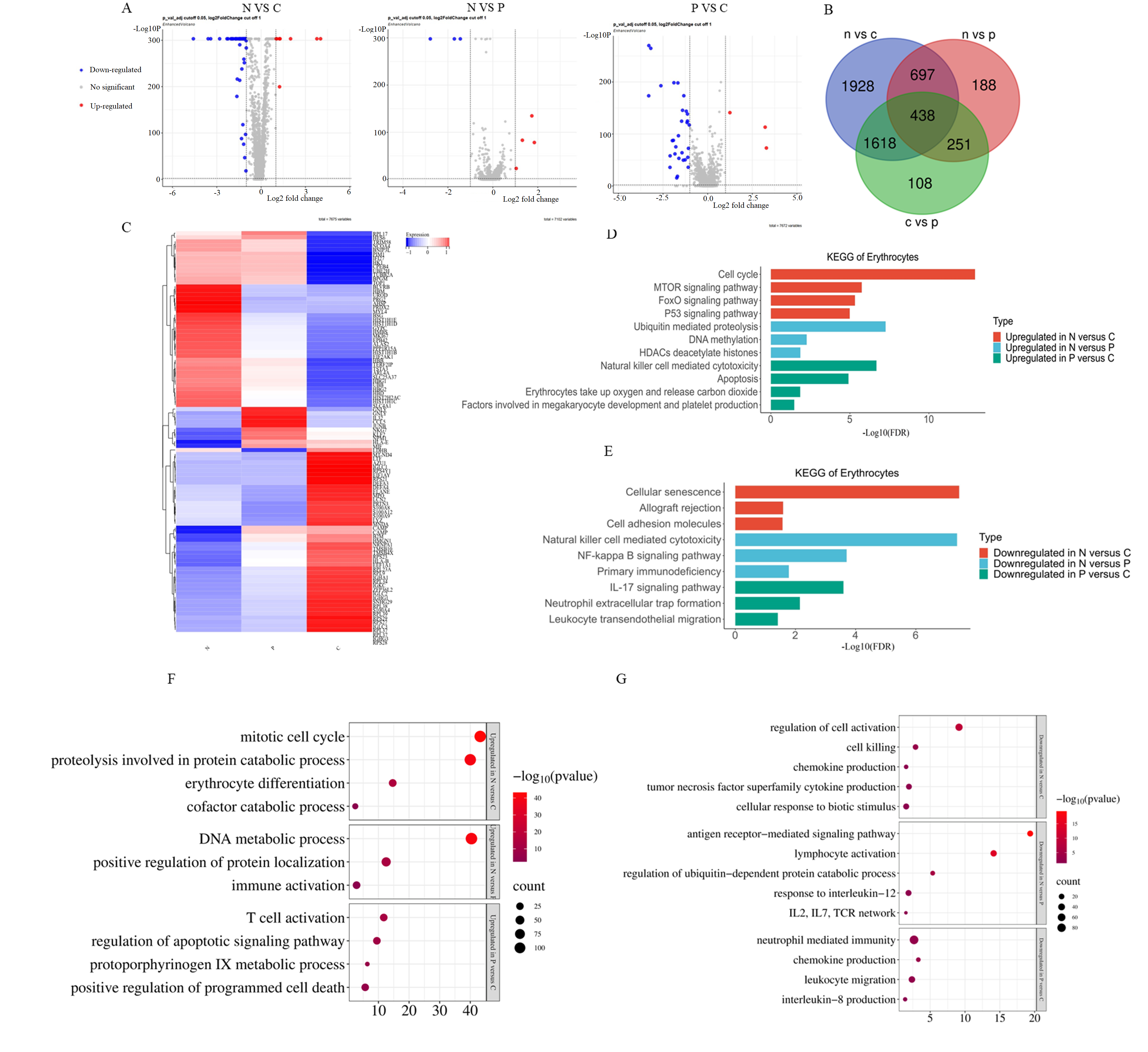

Supplement: Supplementary file 4 — Supporting Information [file CTM2-14-e1671-s007.tif]
