# Supplementary material for: Single‐cell sequencing reveals alterations in the differentiation of bone marrow haematopoietic cells in patients with paroxysmal nocturnal haemoglobinuria
Source: Clin Transl Med. 2024 Jun 25;14(7):e1671. doi: 10.1002/ctm2.1671 (PMC11199056; doi:10.1002/ctm2.1671)
Supplement: Supplementary file 9 — Supporting Information [file CTM2-14-e1671-s003.docx]

**Methods of data analysis**

The single cell sequencing was performed 10× genomics platform. CellRanger (version 5.0.1) was used to align the raw sequencing reads to the human reference genome GRCh38 (2020) and extract the count matrix, with default settings. Cells exhibiting fewer than 100 detected genes and those with mitochondrial content exceeding 15% were excluded. Additionally, cells surpassing 6000 detected genes or having more than 60,000 unique molecular identifiers (UMIs) were eliminated to ensure the exclusion of potential doublets. After excluding low-quality cells, the data underwent normalization, and top 2000 variably expressed genes were identified using the FindVariableFeatures function in Seurat. Principal component analysis (PCA) was applied to reduce the dimensionality of the scRNA-Seq dataset. Cell clusters were determined using the FindClusters function in Seurat with a resolution of 0.3. These clusters were visualized using uniform manifold approximation and projection (UMAP) plots.

To further investigate changes in HSPCs, the cell type labeled as HSPC in Figure 1C1 was extracted using the subset function in Seurat and subsequently reclustered. To provide more context, this involved isolating the HSPC population from the original dataset and subjecting it to a finer level of clustering using Seurat's recluster function.

In our analysis, we used the Harmony package (version 1.0) to perform the batch correction and ensure a robust comparison across datasets. Comparisons for multiple groups were conducted using two-way ANOVA with Sidak’s multiple comparisons test (SPSS, version 17.0). The criterion of statistical significance was set as *p* value less than 0.05.
