# Supplementary material for: Single‐cell sequencing reveals alterations in the differentiation of bone marrow haematopoietic cells in patients with paroxysmal nocturnal haemoglobinuria
Source: Clin Transl Med. 2024 Jun 25;14(7):e1671. doi: 10.1002/ctm2.1671 (PMC11199056; doi:10.1002/ctm2.1671)
Supplement: Supplementary file 10 — Supporting Information [file CTM2-14-e1671-s001.docx]

Figure S1. **Detection of bone marrow hematopoietic cells in 3 PNH patients by flow cytometry.** A. The frequency of CD59- and CD59+ cells in each lineage. B. Grouping of bone marrow hematopoietic cells through CD45 and SSC gating. C-L. CD59 detection on stem cells (C), T cells (D), B cells (E), NK cells (F), neutrophils (G), monocygtes (H), macrophages (I), erythrocytes (J), DCs (K) and megakarcytes (L).

Figure S2. **The changes of T cells in PNH patients compared with normal controls.** A. Volcano plot showing differentially expressed genes (DEGs) of T cells in the three groups. B. Venn diagram showing the summary of DEGs detected by pair-wise comparison at three groups. C. Heatmap displaying the expression of some DEGs of T cells in the three groups based on significance and logFC values. D. GO enrichment analysis of the up-regulated genes (D1) and down-regulated genes (D2) in T cells within three groups. E. KEGG enrichment analysis of the up-regulated genes (E1) and down-regulated genes (E2) in T cells within three groups.

Figure S3. **The changes of NK cells in PNH patients compared with normal controls.** A. Volcano plot showing differentially expressed genes (DEGs) of NK cells in the three groups. B. Venn diagram showing the summary of DEGs detected by pair-wise comparison at three groups. C. Heatmap displaying the expression of some DEGs of NK cells in the three groups based on significance and logFC values. D. GO enrichment analysis of the up-regulated genes (D1) and down-regulated genes (D2) in NK cells within three groups. E. KEGG enrichment analysis of the up-regulated genes (E1) and down-regulated genes (E2) in NK cells within three groups.

Figure S4. **The changes of eryghrocytes in PNH patients compared with normal controls.** A. Volcano plot showing differentially expressed genes (DEGs) of eryghrocytes in the three groups. B. Venn diagram showing the summary of DEGs detected by pair-wise comparison at three groups. C. Heatmap displaying the expression of some DEGs of eryghrocytes in the three groups based on significance and logFC values. D. GO enrichment analysis of the up-regulated genes (D1) and down-regulated genes (D2) in eryghrocytes within three groups. E. KEGG enrichment analysis of the up-regulated genes (E1) and down-regulated genes (E2) in eryghrocytes within three groups.
